# Unveiling the pathogenic mechanisms of NPR2 missense variants: insights into the genotype-associated severity in acromesomelic dysplasia and short stature

**DOI:** 10.3389/fcell.2023.1294748

**Published:** 2023-11-23

**Authors:** Sally Badawi, Divya Saro Varghese, Anjana Raj, Anne John, Hamda S. Al-Musafir, Ahmed J. Al-Ghamari, Alreem R. Alshamsi, Sara H. Ouda, Ghayth Al-Dirbashi, Bassam R. Ali

**Affiliations:** ^1^ Department of Genetics and Genomics, College of Medicine and Health Sciences, United Arab Emirates University, Al-Ain, United Arab Emirates; ^2^ ASPIRE Precision Medicine Research Institute Abu Dhabi, United Arab Emirates University, Al-Ain, United Arab Emirates

**Keywords:** Acromesomelic Dysplasia Maroteaux type (AMDM), cyclic guanosine monophosphate (cGMP), natriuretic peptide receptor 2 (NPR2), protein quality control, short stature

## Abstract

**Introduction:** Natriuretic peptide receptor 2 (NPR2 or NPR-B) plays a central role in growth development and bone morphogenesis and therefore loss-of-function variations in NPR2 gene have been reported to cause Acromesomelic Dysplasia, Maroteaux type 1 and short stature. While several hypotheses have been proposed to underlie the pathogenic mechanisms responsible for these conditions, the exact mechanisms, and functional characteristics of many of those variants and their correlations with the clinical manifestations have not been fully established.

**Methods:** In this study, we examined eight NPR2 genetic missense variants (p.Leu51Pro, p.Gly123Val, p.Leu314Arg, p.Arg318Gly, p.Arg388Gln, p.Arg495Cys, p.Arg557His, and p.Arg932Cys) Acromesomelic Dysplasia, Maroteaux type 1 and short stature located on diverse domains and broadly classified as variants of uncertain significance. The evaluated variants are either reported in patients with acromesomelic dysplasia in the homozygous state or short stature in the heterozygous state. Our investigation included the evaluation of their expression, subcellular trafficking and localization, N-glycosylation profiles, and cyclic guanosine monophosphate (cGMP) production activity.

**Results and Discussion:** Our results indicate that variants p.Leu51Pro, p.Gly123Val, p.Leu314Arg, p.Arg388Gln have defective cellular trafficking, being sequestered within the endoplasmic reticulum (ER), and consequently impaired cGMP production ability. Conversely, variants p.Arg318Gly, p.Arg495Cys, and p.Arg557His seem to display a non-statistically significant behavior that is slightly comparable to WT-NPR2. On the other hand, p.Arg932Cys which is located within the guanylyl cyclase active site displayed normal cellular trafficking profile albeit with defective cGMP. Collectively, our data highlights the genotype-phenotype relationship that might be responsible for the milder symptoms observed in short stature compared to acromesomelic dysplasia. This study enhances our understanding of the functional consequences of several NPR2 variants, shedding light on their mechanisms and roles in related genetic disorders which might also help in their pathogenicity re-classification.

## 1 Introduction

The natriuretic peptide system is a major hormonal pathway that plays a significant role in maintaining blood pressure, keeping balance of body fluids, and regulating cardiovascular homeostasis (Silver, 2006). Among the key components of this system is the natriuretic peptide receptor 2 (NPR-B) encoded by *NPR2* gene, the primary receptor for C-type natriuretic peptide (CNP). CNP is a paracrine regulator of the growth plate and the ligand of the transmembrane receptor protein NPR2. CNP/NPR2 signaling activates the production of cyclic guanosine monophosphate (cGMP) that plays a critical role in endochondral ossification, which is responsible for longitudinal growth in limbs and vertebrae (Potter et al., 2006; Potter, 2011b). In addition to its extracellular and transmembrane domains, NPR2 consists of an intracellular domain with a guanylyl cyclase activity. The latter belongs to a family of membrane-bound enzymes that catalyze the conversion of intracellular guanosine triphosphate (GTP) to cGMP (Potter, 2011a).

Various human genetic disorders have been linked to genetic variations in *NPR2* gene. The most prominent is Acromesomelic Dysplasia 1, Maroteaux type (AMDM1, MIM # 602875), which is inherited in an autosomal recessive manner and mainly caused by loss-of-function (LOF) mutations in this gene (Bartels et al., 2004). AMDM1 is a rare skeletal disorder characterized by distinctive facial features, abnormal limb development, and disproportionate shortening in the limbs and skeletal elements (Langer and Garrett, 1980). Additionally, gain-of-function (GOF) mutations in *NPR2* gene have been associated with the autosomal dominant disorder, epiphyseal chondrodysplasia, Miura type (ECDM, MIM # 615923), characterized by overgrowth due to excessive production of cGMP in the cells (Miura et al., 2012). Furthermore, certain *NPR2* variations in the heterozygous state have been associated with short stature with nonspecific skeletal abnormalities (SNSK, MIM # 616255) (Olney et al., 2006; Vasques et al., 2013).

Several hypotheses have been proposed to elucidate the underlying pathogenic mechanisms caused by *NPR2* genetic variants. One hypothesis implicates in various mutations that might hinder the proper trafficking of NPR2 receptor to the plasma membrane. These variants cause the receptor to be retained within the endoplasmic reticulum (ER) due to improper protein folding and failure to pass the ER folding quality control (Chen et al., 2005; Hume et al., 2009; Brodsky, 2012; Sun and Brodsky, 2019). The latter ultimately results in loss of function and hence decreased ligand binding capacity at the cellular level (Hume et al., 2009; Hisado-Oliva et al., 2015; Li et al., 2022). Another suggested mechanism for some variants that may cause ligand-induced conformational changes and consequently the loss of NPR2 guanylyl cyclase catalytic activity and its ability to produce cGMP (Hachiya et al., 2007).

*NPR2* missense variants have displayed a broad-spectrum of phenotypic heterogeneity among patients with AMDM1 and short stature (Hisado-Oliva et al., 2015; Hanley et al., 2020; Wu et al., 2022). In a recent analysis performed on patients with compound heterozygous and heterozygous carriers of *NPR2* mutations linked to AMDM1 and short stature, respectively, Hanley et al. proposes a genotype-phenotype association of these variants that might be linked to the disease progression (Hanley et al., 2020). However, current literature lacks conclusive associations between the genotype of NPR2 mutations carriers and their subsequent phenotypes and the functional implications for most of these variations have not been studied. There is, therefore, a need to carry out detailed functional validations and clinical analysis on these variants.

**FIGURE 1:**
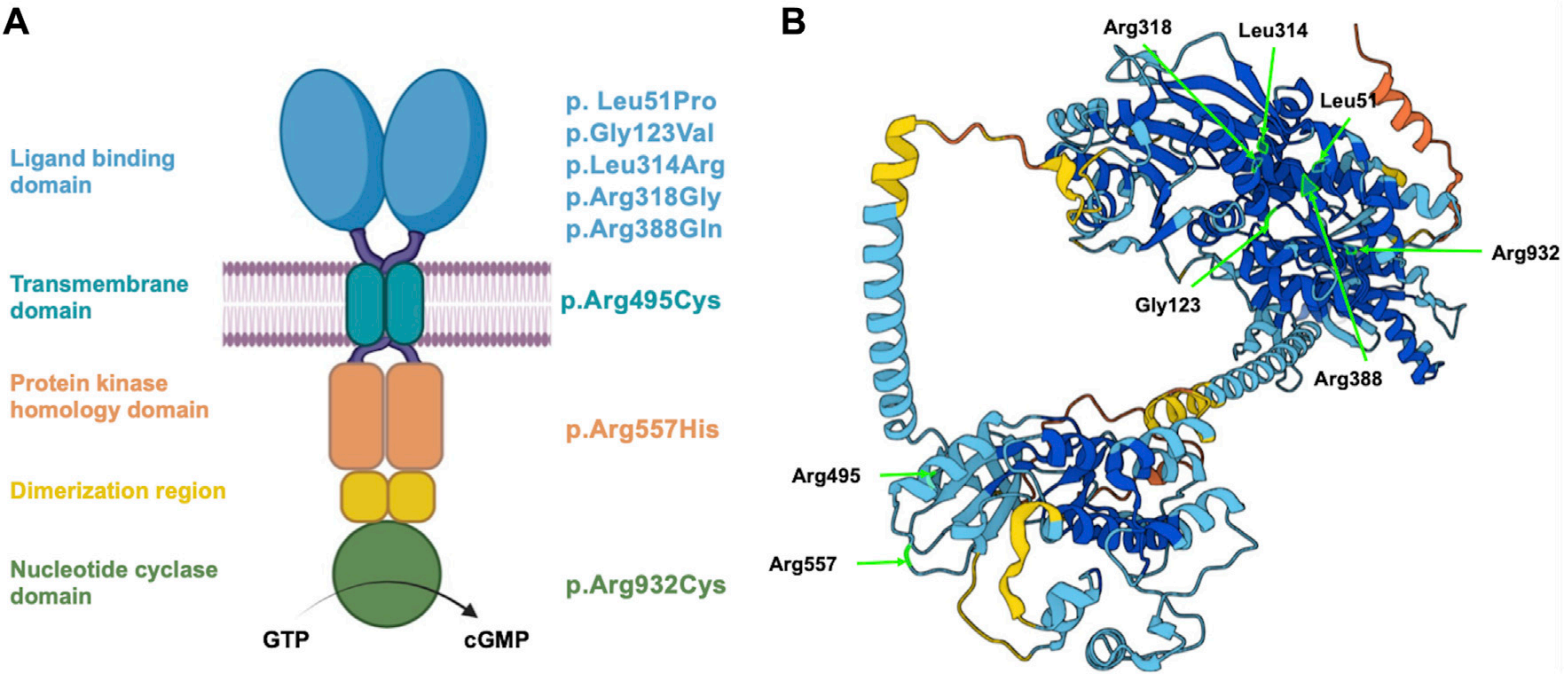
Schematic representation of NPR2 structure and variant localization. **(A)** A schematic diagram displaying the different domains of human NPR2 protein, including the ligand binding, transmembrane, protein kinase homology, and nucleotide cyclase domains. Substituted amino acid residues on the right correspond to the studied coding variants, distributed based on their domain. **(B)** 3D structure of NPR2 protein created in AlphaFold Monomer v2.0 pipeline. Light residues labeled in light green correspond to the substituted amino acids that were evaluated in the manuscript.

In this study, we investigated the effect of eight different *NPR2* missense genetic variations that have been reported to cause variable phenotypes and span the various domains of NPR2 protein as shown in Figures 1A, B. These variants were either homozygous mutations associated with AMDM1, or heterozygous linked to short stature (Table 1). We generated these eight variants by site directed mutagenesis in a mammalian expression vector and examined their impact on NPR2 protein expression, subcellular trafficking and localization, N-glycosylation profiles, and their enzymatic functionality via cGMP production capability. Altogether, our data highlights the potential genotype-phenotype link that might explain the various symptoms observed in short stature and Acromesomelic Dysplasia Maroteaux type 1, as well as provide further evidence of the different mechanism of actions that contribute to the variants’ pathogenicity and clinical severity.

**TABLE 1:** NPR2 missense variants *in silico* predictions.

NPR2 variant	Amino acid substitution	dbSNP	Associated disease	Zygosity state	gnomAD allele frequency	VarSome prediction	Franklin prediction ACMG guidelines	Franklin ACMG classification	wInterVar prediction	Revel prediction	SIFT Prediction	MutationTaster predictions
c.152T>C	p.Leu51Pro	NA	AMDM1 (Tran et al., 2019)	Homozygous	NA	Likely pathogenic	VUS	PM2, PP3, PP2	VUS	Deleterious: Supporting Pathogenic	Damaging	Disease causing
c.368G>T	p.Gly123Val	rs763502307	AMDM1 (Ain et al., 2019)	Homozygous	0.000003976	Likely pathogenic	VUS	PP3, PM2, PP2	VUS	Deleterious: Moderate Pathogenic	Damaging	Disease causing
c.941T>C	p.Leu314Arg	NA	AMDM1 (Irfanullah et al., 2018)	Homozygous	NA	VUS	VUS	PM2, PP2	VUS	Uncertain	Tolerated	Disease causing
c.952C>G	p.Arg318Gly	rs144940095	Short stature (Andrade et al., 2022)	Heterozygous	0.00008839	VUS	VUS	PM2, PP2	VUS	Uncertain	Tolerated	Disease causing
c.1163G>A	p.Arg388Gln	rs1828106198	AMDM1 (Amano et al., 2020)	Homozygous	NA	VUS	VUS	PM2, PP3, PP2	VUS	Deleterious: Supporting Pathogenic	Damaging	Disease causing
c.1483C>T	p.Arg495Cys	rs1436133890	Short stature (Hwang et al., 2020)	Heterozygous	0.000003978	VUS	VUS	PM2, PP2	VUS	Benign: Supporting	Damaging	Disease causing
c.1670G>A	p.Arg557His	rs766118843	Short stature (Plachy et al., 2020)	Heterozygous	0.00001195	VUS	VUS	PM2, PP2	VUS	Benign: Supporting	Damaging	Disease causing
c.2794C>T	p.Arg932Cys	rs760285654	Short stature (Hauer et al., 2018)	Heterozygous	0.000003983	VUS	Likely pathogenic	PS4, PM2, PP2, PP5	VUS	Uncertain	Damaging	Disease causing

VUS: variant of unknown significance, AMDM1: Acromesomelic dysplasia Maroteaux-type 1, PM2: pathogenic moderate, PP2/PP3/PP5: pathogenic supporting, PS5: pathogenic strong, NA: not available.

## 2 Materials and methods

### 2.1 Pathogenicity *in silico* prediction of the eight studied NPR2 missense variants

Considering the clinical reports and the most recent evidence curated in the Human Gene Mutation Database (HGMD), that associate *NPR2* variants to AMDM1 and short stature at the time of our study, eight missense variants were chosen to be investigated. It is worth noting that these selected variants detailed in Table 1, were selected to be structurally located in the various functional domains across the NPR2 protein as illustrated in Figures 1A, B, with four of them causing AMDM1 and four reported to be responsible for short stature. In a previous work, we have shown that 11 of 12 studied missense mutants causing AMDM1 are retained in the ER (Hume et al., 2009); and we wanted from this work, in addition to further confirming the involvement of ERAD in the mechanisms of recently reported AMDM1-causing mutation, to examine if this ER retention is also underlying the mechanism of some of the short stature causing mutations. Although not necessarily proportionally, mutations at the major domains were represented in this selected list with mutations in the largest domain “the ligand binding domain” represented with five mutations. To assess the effect of the eight *NPR2* missense variants structurally located in the various domains of NPR2 structure, different *in silico* prediction tools have been used. Clinical significance of the variants was predicted using VarSome.com (https://varsome.com) software (accessed in October 2023), Franklin by genoox (https://franklin.genoox.com/clinical-db/home) software (accessed in June 2023) and wInterVar (https://wintervar.wglab.org) software (accessed in November 2023). Allele frequencies of these variants was obtained from gnomAD database v2.1.1 (https://gnomad.broadinstitute.org). Sorting Intolerant from Tolerant (SIFT) algorithm was used to predict the effect of the studied variants on NPR2 protein function based on the physiochemical similarity and sequence homology between variants and WT (https://sift.bii.a-star.edu.sg). Additionally, to predict the pathogenicity of NPR2 variants, Revel and MutationTaster (https://www.mutationtaster.org/MutationTaster69/index.html) software were used. All the latter software used for these predictions were accessed as of June 2023. Variants were considered possibly damaging if they were found to affect NPR2 protein structure in at least one of the tested tools, otherwise, it is considered benign.

### 2.2 Primer’s design and site directed mutagenesis for generation of NPR2 missense variants

The construction and characterization of the HA-tagged human *NPR2* wild-type plasmid (HA-tagged NPR-B) has been described by us previously (Hume et al., 2009). The transcript used for *NPR2* is the protein coding transcript NM_003995.4. Using the wild-type construct as a template, we have generated the 8 missense variants (p.Leu51Pro, p.Gly123Val, p.Leu314Arg, p.Arg318Gly, p.Arg388Gln, p.Arg495Cys, p.Arg557His, and p.Arg932Cys) by the Quick-Change site-directed mutagenesis kit with the *Pfu* Ultra High-Fidelity DNA polymerase (Stratagene). The primers used to this mutagenesis are listed in Supplementary Table S1 and were designed using PrimerX software (https://www.bioinformatics.org/primerx/) and were custom-made by Metabion International AG (https://www.metabion.com/). The generation of the correct variants were confirmed by the dideoxy Sanger DNA sequencing using the ABI 3130xl automated fluorescent Genetic Analyzer (Applied Biosystems). Sequences were aligned using Crustal Omega software (https://www.ebi.ac.uk/Tools/msa/clustalo/).

### 2.3 Cell culture and transfection

HEK293T and HeLa cells were cultured in Dulbecco’s Modified Eagle Medium (Gibco) supplemented with 10% fetal bovine serum (Gibco), and antibiotic–antimycotic (Gibco) at 37 °C and 5% CO_2_ as recently described (Badawi et al., 2022). Cells were grown in 6-well and 24-well tissue culture plates. Transfection of WT and variant generated plasmids was performed using FuGENE HD transfection reagent (Promega), according to manufacturer’s protocol.

### 2.4 Immunoprecipitation and SDS-PAGE immunoblotting

HEK293T cells seeded into 6-wells plates were transfected with HA-tagged WT or mutants *NPR2*. 48 h post transfection, cells were harvested, and lysed using IP lysis buffer (Pierce Inc.) based on the manufacturer’s protocol containing protease inhibitor cocktail (Pierce Inc.). Total lysates were quantified using colorimetric bicinchoninic acid protein assay (BCA kit, Pierce Inc.). HA-tagged NPR2 were immunoprecipitated with anti-HA agarose beads (Pierce Inc.). Briefly, total lysates were incubated overnight at 4 °C with rotation. Immunoprecipitates were then collected by centrifugation, and then used for expression and enzymatic analysis assays. Total protein lysates and immunoprecipitates were resolved on 4%–12% precast gradient gel (GeneScript) and transferred into PVDF membrane (Thermo Fisher Scientific). Membranes were then probed with the corresponding primary antibodies: anti-HA (1:2000 Cell Signaling Technology, cat# 2367S), anti-GAPDH antibody (1:1000 Cell Signaling Technology, cat# 2118S) and their respective secondary antibodies (Sigma-Aldrich). Enhanced Chemiluminescence Plus reagent (Pierce) was used for signal detection using the Typhoon FLA 9500 imager (GE Healthcare Biosciences, Piscataway, NJ, United States). Blot analysis quantification was then performed using ImageJ software (Schneider et al., 2012).

### 2.5 Immunofluorescence and confocal microscopy

HeLa cells were seeded on sterile cover slips and 24 h later, cells were co-transfected with HA-WT or HA-mutant *NPR2* plasmids and the plasma membrane marker, GFP-tagged *HRas* plasmid. Twenty-four hours post transfection, cover slips were washed three times with phosphate-buffered saline (PBS) and then fixed with ice cold methanol at −20 °C for 5 min. Cells were then blocked with 3% bovine serum albumin (BSA) (Sigma-Aldrich) at room temperature for 30 min. Fixed cells were then stained with Anti-HA primary antibody (Santa Cruz, cat# sc-7392) and anti-Calnexin (Cell Signaling Technology, cat# 2368) in a dark chamber for 1 h at room temperature. Cover slips were then washed three times with PBS and then incubated with the respective secondary antibody (Thermo Fischer Scientific) for 45 min in dark at room temperature. Cells were then washed again three times with PBS and mounted with immunofluor medium (ICN Biomedicals). Images were acquired using Nikon confocal Eclipse 80I microscope (Nikon Instruments Inc.) using the ×100 objective and images were later merged and analyzed using ImageJ software.

### 2.6 Analysis of the NPR2 N-glycosylation profiles

HA-tagged NPR2 immunoprecipitates were subjected to enzymatic treatment using Peptide-N-glycosidase F (PNGase F) (New England Biolabs) or with endoglycosidase H (Endo H) (Sigma-Aldrich), to remove N-linked oligosaccharides from the NPR2 protein localized in various cellular compartments. These assays can discriminate between the ER immature form and the post ER mature form of the protein. Briefly and based manufacturer’s protocols, immunoprecipitates were denatured using denaturation buffer with heating at 100 °C. After adding the required reaction buffers and the corresponding enzymes, reaction mixtures were then incubated for 3 h and 1 h at 37 °C with PNGase F and Endo H, respectively.

### 2.7 Enzyme-Linked Immunosorbent Assay for the determination of cGMP production activity

HEK293T cells were seeded into 12-well plates and transfected with HA-WT and HA-mutants *NPR2* plasmids. 48 h post transfection, cells were treated with C natriuretic peptide (CNP) substrate (TOCRIS, 100 nM) dissolved in serum free-DMEM media for 10 min at 37 °C. To stop the phosphodiesterase activity and stabilize cGMP, 0.1 M HCL was added to the cells and incubated for 10 min at room temperature. Cell lysates were then collected and cGMP Enzyme-Linked Immunosorbent Assay (ENZO Life Sciences, Madison Avenue, NY, United States, cat# ADI-900-164) was used to measure cGMP levels.

### 2.8 Statistical analysis

Data were analyzed using GraphPad Prism version 10.0.0 and displayed as mean ± standard error (SE). Experiments were conducted at least three times and data were analyzed using the ordinary one-way ANOVA test with Dunnet’s test for multiple comparison. *p*-value <0.05 was considered significant.

## 3 Results

### 3.1 In silico prediction analysis of *NPR2* variants

All the eight studied *NPR2* variants are of extremely low allele frequency in gnomAD database (Table 1). Additionally, the majority of these variants have been clinically classified as variants of unknown significance using VarSome.com, wInterVar, and Franklin by genoox databases based on the classification guidelines by the American College of Medical Genetics and Genomics (ACMG) with the exception of p.Leu51Pro, p.Gly123Val (rs763502307), and p.Arg932Cys (rs760285654) that are classified as likely pathogenic by VarSome.com and Franklin software (Table 1). Furthermore, pathogenicity analysis using different prediction tools including REVEL, SIFT, and MutationTaster, has shown that all these eight variants were predicted to be possibly damaging by at least one of the prediction tools used in this analysis. Altogether this *in silico* analysis suggests the necessity of functional validation of *NPR2* missense variants to further characterize their pathogenicity and thus update their clinical significance status.

### 3.2 Cellular trafficking and localization suggest that variants p.Leu51Pro, p.Gly123Val, p.Leu314Arg and p.Arg388Gln are retained in the ER presumably via the ER-associated protein degradation machinery

**FIGURE 2:**
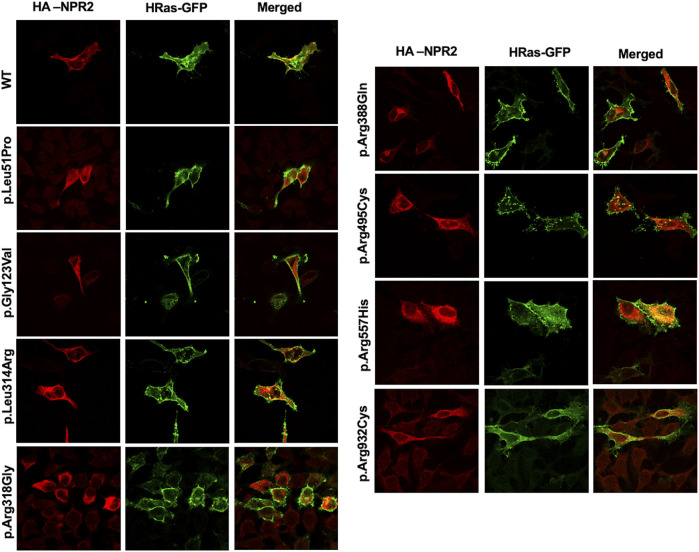
WT and variants NPR2 subcellular localization using plasma membrane marker. HA-tagged WT and variants NPR2 plasmids were transiently cotransfected with GFP‐tagged HRas (plasma membrane marker) into HeLa cells for 24 h. Cells were then fixed and stained with anti-HA antibody. Images were captured using the ×100 oil immersion objective of the NIKON Eclipse Confocal Microscope equipped with FITC and TRITC filters. Scale bar = 50 μm.

**FIGURE 3:**
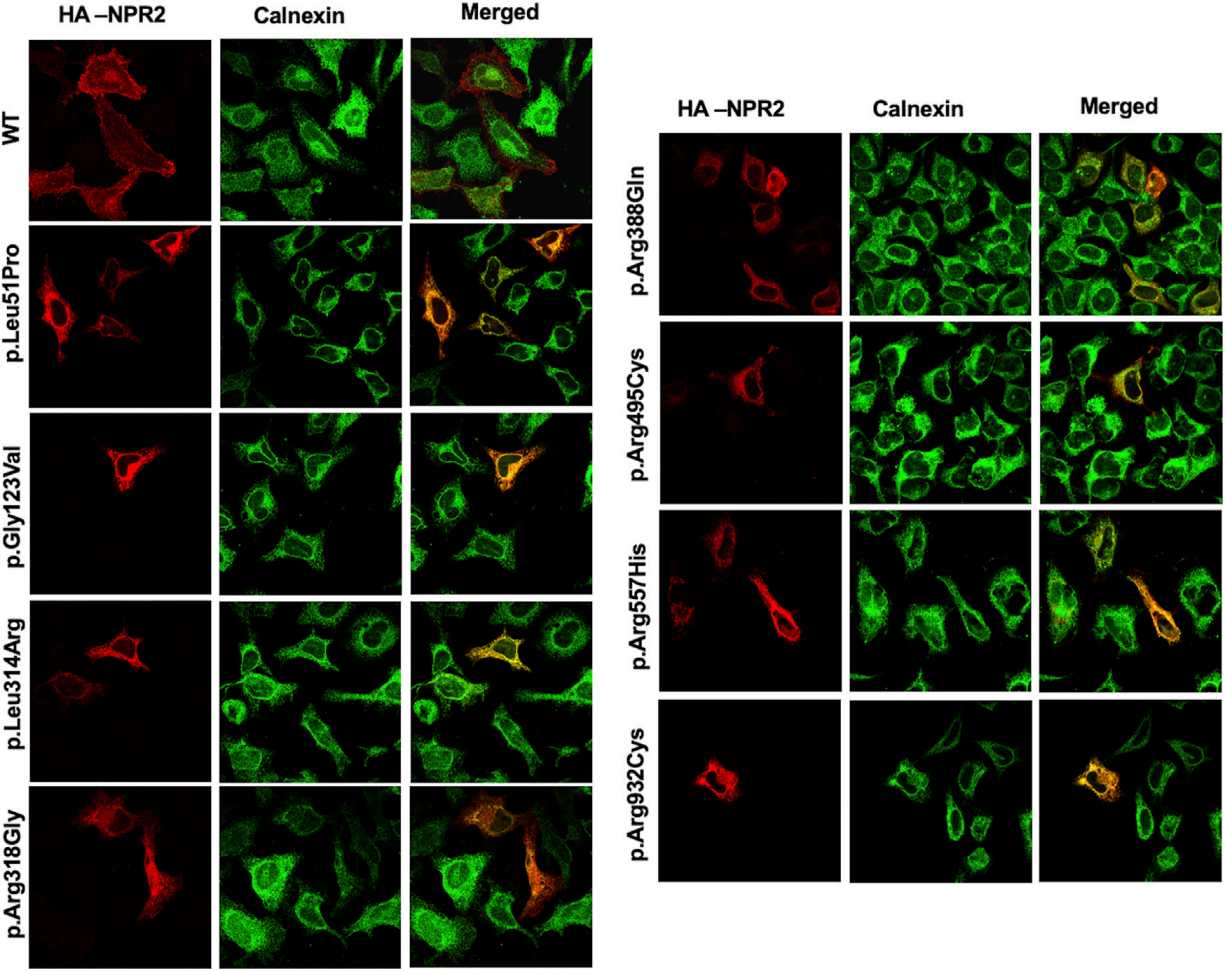
WT and variants NPR2 subcellular localization using endoplasmic reticulum marker. HA-tagged WT and variants NPR2 plasmids were transiently transfected into HeLa cells for 24 h. Cells were then fixed and stained with anti-HA and anti-Calnexin (Endoplasmic reticulum marker) antibodies. Images were captured using the ×100 oil immersion objective of the NIKON Eclipse Confocal Microscope equipped with FITC and TRITC filters. Scale bar = 50 μm.

To assess the intracellular localization of *NPR2* variants, HA-tagged *NPR2* and GFP-tagged *HRas* plasmids were co-transfected into HeLa cells. As shown in the confocal microscopy images (Figures 2, 3; Supplementary Figure S1), HA-tagged WT-NPR2 displayed a heterogenous localization pattern, wherein it localizes with the plasma membrane marker, GFP-tagged *HRas* and some with the ER specific marker, calnexin. The fraction that co-localizes with the ER marker is presumably the in-transit phase and/or the fraction of the WT protein that failed to fold properly. In contrast, four NPR2 variants p.Leu51Pro, p.Gly123Val, p.Leu314Arg and p.Arg388Gln, displayed exclusively an endoplasmic reticulum pattern by co-localizing with calnexin as shown in Figures 2, 3, without any overlap with the plasma membrane marker suggesting their retention by the ER associated protein degradation machinery. Moreover, the other variants, p.Arg318Gly, p.Arg495Cys, p.Arg557His, and p.Arg932Cys appeared to display heterogenous localizations, where they exhibited both an ER and plasma membranal localization profiles (Supplementary Figure S1).

### 3.3 The N-glycosylation profiling confirms the confocal imaging of p.Leu51Pro, p.Gly123Val, p.Leu314Arg, and p.Arg388Gln retention in the ER

**FIGURE 4:**
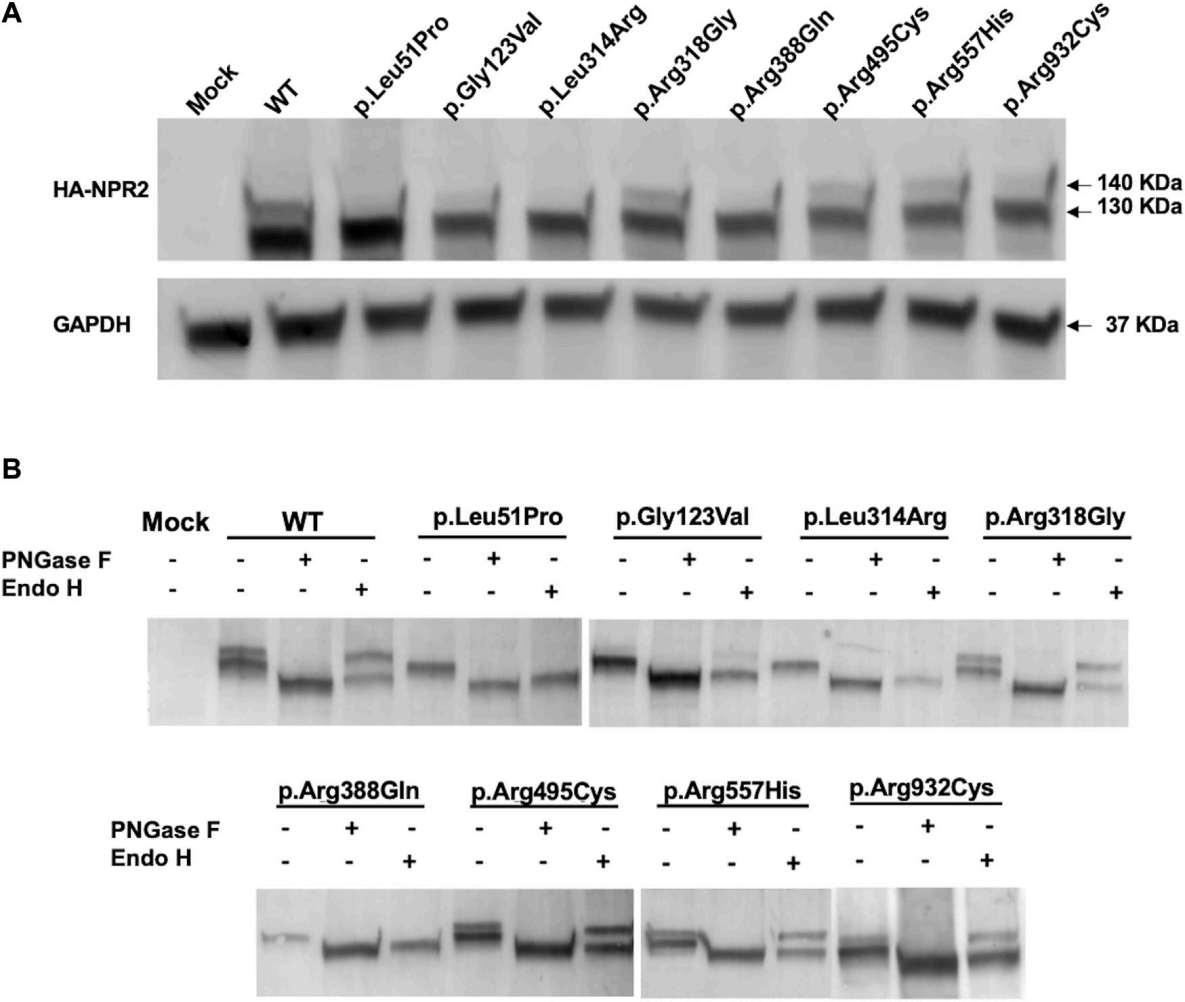
Exogenously expressed WT and variants NPR2 expression profiles. **(A)** Immunoblot of exogenously expressed HA-tagged NPR2 protein. WT and NPR2 variants transiently transfected in HEK293T cells for 48 h. Anti-HA primary antibody was used to stain NPR2 protein. GAPDH was used as a loading control. **(B)** HA-tagged proteins immunoprecipitated using HA-epitope agarose resins were digested using PNGase F and EndoH enzymes for 1 h and 3 h, respectively. Treated lysates were then analyzed by SDS-PAGE using anti-HA antibody. Mock sample represent untransfected control cells.

To further validate the immunofluorescence results and investigate whether the studied *NPR2* missense variants impact expression and localization, HA-tagged WT and mutants NPR2 were exogenously expressed in HEK293T cellular model. Immunoblotting analysis by SDS-PAGE shows that total lysates of overexpressed WT-NPR2 consists of two molecular weight protein bands with the upper band presumably representing the mature form and the lower band the immature ER-located fraction (one at ∼130 and one at ∼140 KDa) (Figure 4A). It seems that even NPR2 WT does not mature quantitatively with a significant fraction failing to achieve full maturation suggesting retention in the ER. Similarly, variants p.Arg318Gly, p.Arg495Cys, p.Arg557His, and p.Arg932Cys showed both lower and upper bands in comparison to the WT, with varying ratios between the two bands. In contrast, variants p.Leu51Pro, p.Gly123Val, p.Leu314Arg, and p.Arg388Gln showed only the lower molecular weight immature band at ∼130 KDa suggesting their quantitative retention in the ER presumably by ERAD.

To further confirm this, we conducted glycosides sensitivity and resistance assays. Enzymatic analysis using glycosidases assays showed that treatment with Peptide-N-glycosidase F (PNGase F) that aims to digest all N-linked oligosaccharides regardless of the complexity of their N-glycans resulted in the digestion of both NPR2 bands into a lower molecular weight band (∼125 KDa) for the WT and all the mutants, indicating that both bands correspond N-linked glycoprotein whether they are residing in the ER (immature form) or located on the plasma membrane (mature form). Crucially, treatment with Endoglycosidase H (Endo H), an assay designed to discriminate between the mature and immature forms of glycoproteins, resulted in a shift in the mobility of the lower molecular weight immature band only of the WT and the variants corresponding to the immature N-linked NPR2 protein residing in the endoplasmic reticulum (Figure 4B). The protein bands of p.Leu51Pro, p.Gly123Val, p.Leu314Arg, and p.Arg388Gln variants have been shifted quantitatively to the lower band (∼125 KDa) confirming their immature status and exclusive ER localization and hence further confirming retention by ERAD. Variants p.Arg318Gly, p.Arg495Cys, p.Arg557His, and p.Arg932Cys exhibited a behavior similar to WT, except for p.Arg495Cys, p.Arg557His, and p.Arg932Cys variants showing more of the immature band which may reflect partial ER retention.

### 3.4 Enzymatic functional characterization of NPR2 receptor activity suggest the total loss of function of the four ER-retained variants together with the variant structurally located in the guanylate cyclase catalytic domain

**FIGURE 5:**
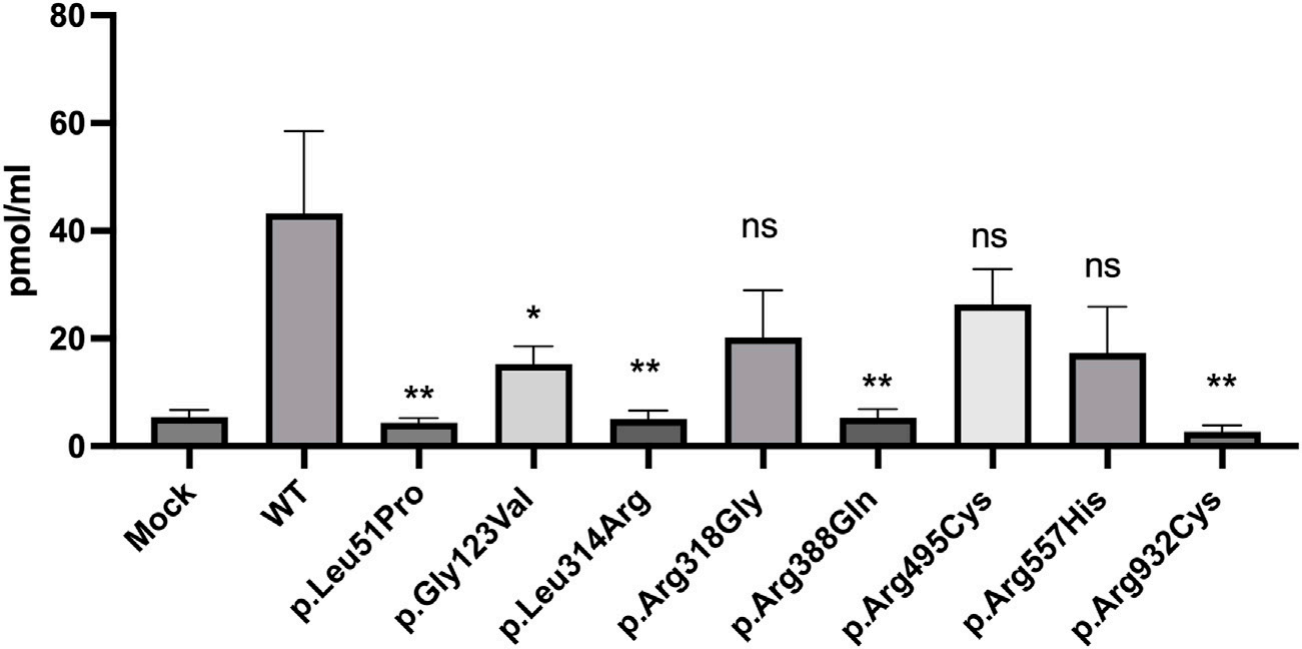
cyclic guanosine monophosphate (cGMP) production by NPR2 variants. A histogram displaying cGMP levels measured by Enzyme linked immunosorbent assay (ELISA) in CNP treated cells transiently transfected with WT or variant NPR2 for 48 h. The graph displays average measurements of five independent experiments. cGMP levels were measured in pmol/mL. *p < 0.05; **p < 0.01; ns: not significant.

To evaluate the functional competency of the studied missense variants of NPR2, the catalytic activities of the guanylyl cyclase activity of WT and the eight variants were measured using competitive ELISA assay (procedure is detailed in the methods section). Cyclic guanosine monophosphate (cGMP) released by HEK293T cells transiently expressing WT or missense variants of NPR2 was evaluated by measuring the catalytic activity of these variants in a C-type natriuretic peptide dependent manner. Transfected cells treated with 100 nM CNP have shown varying cGMP levels compared to the WT. As shown in Figure 5, compared to the WT-NPR2, p.Leu51Pro, p.Gly123Val, p.Leu314Arg, p.Arg388Gln, and p.Arg932Cys displayed quantitative loss of cGMP production with values for these mutants similar to the values obtained with mock transfected cells. The loss-of-function of guanylyl cyclase activity in these variants (p.Leu51Pro, p.Gly123Val, p.Leu314Arg, p.Arg388Gln) correlated with the abnormal retention in the ER. In addition, variant p.Arg932Cys which is located in the catalytic domain also lost its activity quantitively. Furthermore, the other three mutants (p.Arg318Gly, p.Arg495Cys and p.Arg557His) displayed clear reduction in their activities compared to WT but those differences were not statistically significant. It was noticeable that all the variants that displayed reduced cGMP production and abnormal ER retention are associated with AMDM1 whereas the mutants associated with short stature retained some enzymatic activity except for p.Arg932Cys. We therefore could speculate that p.Arg932Cys variant is expected to cause AMDM1 if present in the homozygous state.

## 4 Discussion

In the current study, we explored the effect of four *NPR2* missense variants reported to be associated with AMDM1 disease, namely, p.Leu51Pro, p.Gly123Val, p.Leu314Arg, and p.Arg388Gln. These variants were identified in the homozygous states in patients from various populations (Irfanullah et al., 2018; Ain et al., 2019; Tran et al., 2019; Amano et al., 2020). Substitutions of the studied amino acids are located within the extracellular ligand binding domain of NPR2 protein. Their functional implications were not studied previously and therefore their pathogenicity needed further evidence to elevate their classification from variant of uncertain significance to likely pathogenic or even pathogenic. Our findings provide additional evidence on their pathogenicity suggesting that all four variants were abnormally and almost quantitively retained by the ER associated degradation machinery and failed to traffic to the cell’s plasma membrane. This has been shown to be the case for 11 out of 12 NPR2 mutants that we have evaluated previously (Hume et al., 2009). Furthermore, these variants have exhibited a significant decrease in the production of cGMP, further supporting the *in silico* predictions that suggested their loss of function and consequently disease-causing capabilities. Collectively, these results provide evidence that the pathogenic mechanism underlying AMDM1 in these mutations arises from the improper folding of NPR2 protein leading to its defective trafficking to its intended destination and consequently loss of its function.

While we are among the first to investigate the functionality and pathogenicity of p.Leu51Pro, p.Gly123Val, and p.Leu314Arg, a study by Irfanullah et al. carried out molecular dynamic analysis, *in silico* modeling, and generation of 3D structure of p.Leu314Arg NPR2 variant, suggested that the formation of NPR2 homodimer is affected, leading to a moderate reduction in cGMP production levels (Irfanullah et al., 2018). Moreover, our results show that although cGMP levels were at least 8 folds reduced in comparison to WT-NPR2, it is probably attributed to the incorrect protein folding and defective N-glycosylation profile and retention in the ER. These factors hinder proper NPR2 intracellular trafficking and lead to impaired catalytic activity at the cellular level. Additionally, our results for p.Arg388Gln are in line with Amano et al. functional analysis findings’ indicating that p.Arg388Gln is a loss of function variant associated with AMDM1 clinical phenotype (Amano et al., 2020).

Furthermore, out of the studied heterozygous variants linked to short stature, three variants, namely, p.Arg318Gly, p.Arg495Cys, and p.Arg557His, displayed clear, but not statistically significant in our cellular system, functional behavior compared to WT. The enzymatic activities of these mutants were reduced. These results, reduction but not total loss of their enzymatic activities, are consistent with their subcellular localization and glycosylation profiles as they exhibited some plasma membrane localization and some mature N-glycosylation profiles. It is noteworthy that regardless of their location along NPR2 protein; p.Arg318Gly: ligand binding domain, p.Arg495Cys: transmembrane domain, p.Arg557His: kinase homology domain; these variants displayed lower availability at the cellular membrane and consequently reduced cGMP level after induction with CNP. These variants could probably exhibit a different mechanism of pathogenicity that might explain the milder severity or likely pathogenic evaluation of the clinical phenotype in short stature patients identified with these variants in comparison to others (Hwang et al., 2020; Plachy et al., 2020; Andrade et al., 2022). Moreover, in a study by Hwang et al., authors have shown that treating p.R495C transfected cells with CNP displayed a significant decrease in cGMP level. These slight differences in results might be attributed to the different cellular model that have been used. In our system, we evaluated the subcellular localization, N-glycosylation profiles and enzymatic activities and they are all consistent with partial loss of function. Notably, different cellular models might behave differently in response to exogenously expressed proteins, where Hwang et al. have utilized COS-7 cell line while we have used HEK293T cell line, that are derived from monkeys and humans, respectively. Additionally, while there are some technical differences in the methods used by both, authors have not shown any expression profiles related to p.Arg495Cys in their utilized cellular model. The reported significant decreased activity results that might be due to decreased expression or other technical issues (Hwang et al., 2020). Furthermore, substitution of Arg at residue 557 with Cys instead of His was linked to ADMD1 disease in a compound heterozygous status. Expression of NPR2 p.Arg557Cys was significantly reduced compared to WT but partially colocalized to the plasma membrane and displayed a more severe short stature characteristics (Li et al., 2022). Unlikely, p.Arg932Cys NPR2 variant located in the guanylyl cyclase domain has displayed a similar expression pattern compared to WT-NPR2 but showed a highly significant reduction in cGMP compared to WT NPR2. While the location of this variant in the catalytic domain explains the impaired cGMP production ability, literature still lacks enough description of the clinical characteristics of patients carrying this variant. However, we anticipate that it is highly likely that it will cause AMDM1 if present in the homozygous state.

In our view, the underlying pathogenesis mechanisms of NPR2 missense mutations and their clinical implications (AMDM1, short stature, etc.) are complex and therefore require more detailed investigation. For example, some mutations lead to total loss of NPR2 function when present in the homozygous state (due to their quantitative trafficking defect and retention in the ER, for example,) but will lead to haploinsufficiency and dominant negative effects when present in the heterozygous state with the WT allele. The dominant negative effects of some NPR2 disease-causing mutant alleles on the WT allele have been extensively documented (Vasques et al., 2013; Amano et al., 2014; Hisado-Oliva et al., 2015; Hwang et al., 2020; Wu et al., 2022). On the other hand, some mutants (the ones that traffic normally to the plasma membrane but lost their catalytic activity, for example,) might only cause haploinsufficiency in the heterozygous state and therefore the overall loss of function is lower than those that exert dominant negative effects. In addition, trafficking defects and/or loss of function might not be quantitative for some mutations and therefore their impact will be proportional to that loss. Furthermore, modifier genes might contribute to variability to the clinical presentations. Overall, perhaps we should view the impact of those mutations as a spectrum ranging from total loss of function to mild or no impact for some variants.

In summary, throughout this study that investigates variants linked to both AMDM1 and short stature diseases, we show that different variants in *NPR2* gene might exhibit variable mechanisms of action and impact that contribute to distinct clinical phenotype and severity. Altogether our findings have provided further insights into the functional implications and pathogenicity of various variants associated with two rare growth-related diseases. However, further clinical and genetic studies are required to demonstrate the relation between the zygosity of NPR2 mutations and their clinical manifestations in relation to the diseases especially for short stature-related variants.

## Data Availability

The original contributions presented in the study are included in the article/Supplementary Material, further inquiries can be directed to the corresponding author.
