# Supplementary figures and images for: Unveiling the pathogenic mechanisms of NPR2 missense variants: insights into the genotype-associated severity in acromesomelic dysplasia and short stature
Source: Front Cell Dev Biol. 2023 Nov 23;11:1294748. doi: 10.3389/fcell.2023.1294748 (PMC10702138; doi:10.3389/fcell.2023.1294748)

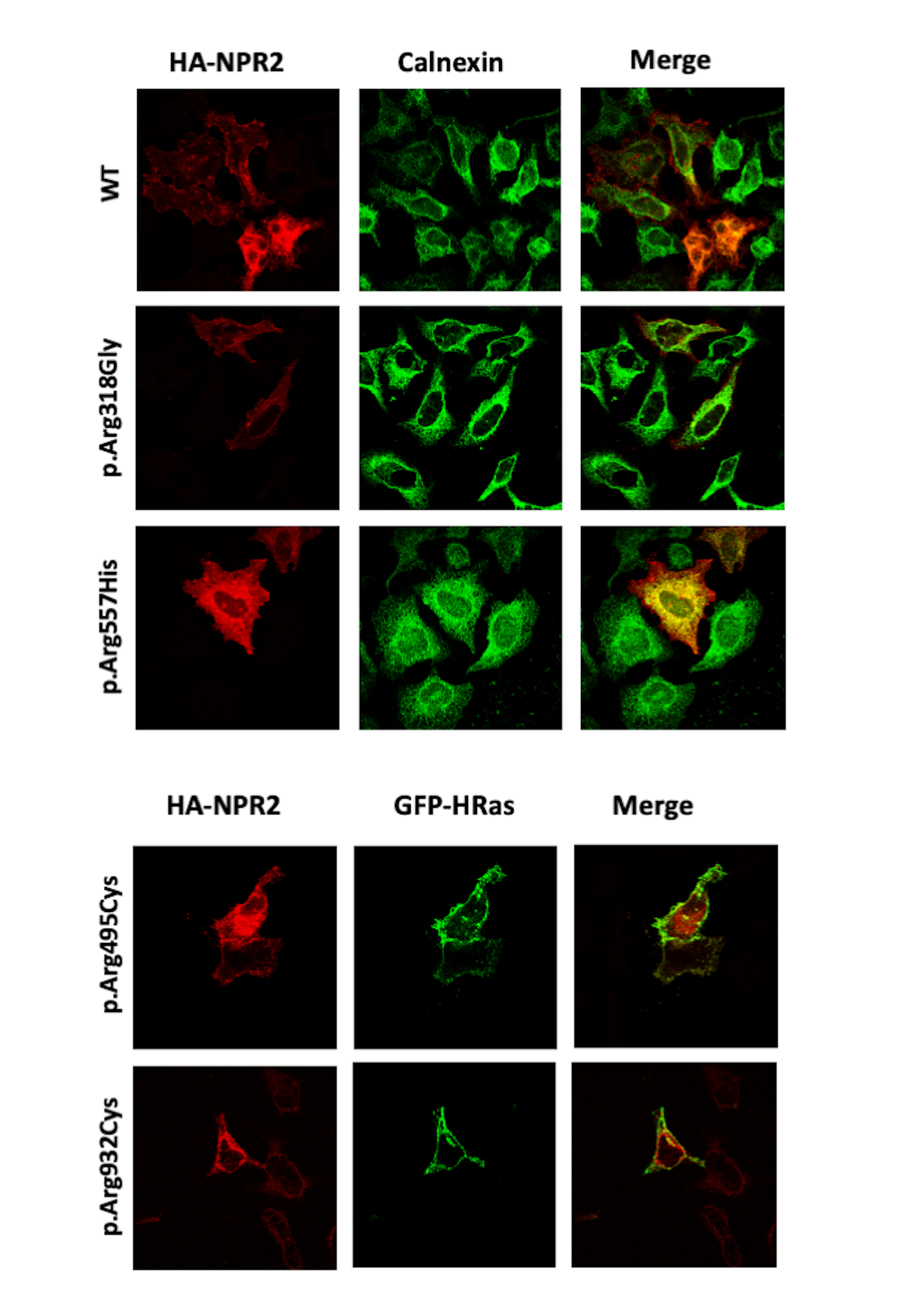

Supplement: Supplementary file 1 [file Image1.TIFF]
