# Supplementary material for: Unveiling the pathogenic mechanisms of NPR2 missense variants: insights into the genotype-associated severity in acromesomelic dysplasia and short stature
Source: Front Cell Dev Biol. 2023 Nov 23;11:1294748. doi: 10.3389/fcell.2023.1294748 (PMC10702138; doi:10.3389/fcell.2023.1294748)
Supplement: Supplementary file 2 [file Table1.DOCX]

**Table** **S1**: NPR2 missense variants designed primers for site directed mutagenesis.

| **NPR2 variant** | **Forward primer** | **Reverse primer** |
| --- | --- | --- |
| WT-NPR2 | 5'-CCG TCC ACC AAA TCT GCT TC-3' | 5-TGT TGG GAG GGT CTA TGC AG-3' |
| p.Leu51Pro | 5'-GAC CCG CTG TGG CAC CAG CTG TGG AGG CTC-3' | 5'-GAG CCT CCA CAG CTG GTG CCA CAG CGG GTC-3' |
| p.Gly123Val | 5'-CTG CTG ACT GCG GTT GCT GTG GCC TC-3' | 5'- GAG GCC ACA GCA ACC GCA GTC AGC AG-3' |
| p.Leu314Arg | 5-CAG AAT CGT CTG CGG ATA AGA GCC CG-3' | 5'-CGG GCT CTT ATC CGC AGA CGA TTC TG-3' |
| p.Arg318Gly | 5'- GCT GAT AAG AGC CGG GGA AGA CTT TGG-3' | 5'-CCA AAG TCT TCC CCG GCT CTT ATC AGC-3' |
| p.Arg388Gln | 5’-GAC AAG AAC AAT GAC GAA GAG ACT GAC TTT GTC -3' | 5’-GAC AAA GTC AGT CTC TTG GTC ATT GTT CTT GTC -3' |
| p.Arg495Cys | 5’-CAT GTT GTG GCG TAT TTG CTG GGA AGA ACT GC-3' | 5’-GCA GTT CTT CCC AGC AAA TAC GCC ACA ACA TG-3' |
| p.Arg557His | 5'-CAT GTG AAT AAG AAG CAC ATT GAG CTG ACC CG-3' | 5'-CGG GTC AGC TCA ATG TGC TTC TTA TTC ACA TG-3' |
| p.Arg932Cys | 5’-CAT GCA CCA GAA ATT GCT TGT ATG GCC CTA GCA TTA C-3' | 5'-GTA ATG CTA GGG CCA TAC AAG CAA TTT CTG GTG CAT G-3' |
